# Editorial: Cancer metabolism: molecular insights, metabolic crosstalk in the tumor microenvironment, and implications for therapy

**DOI:** 10.3389/fonc.2023.1289397

**Published:** 2023-09-18

**Authors:** Balkrishna Chaube, Parmanand Malvi

**Affiliations:** ^1^ Department of Comparative Medicine, Yale Center for Molecular and Systems Metabolism and Vascular Biology and Therapeutics Program, Yale University School of Medicine, New Haven, CT, United States; ^2^ Department of Biochemistry and Molecular Genetics, University of Alabama at Birmingham, Birmingham, AL, United States

**Keywords:** cancer, cancer/immune metabolism, tumor microenvironment, metabolic disorder and cancer, metabolomics, bioenergetics, metabolic imaging

## Introduction

Cancer, as one of the most pressing health issues of the world, continues to be a focal point of extensive research (1). Over the years, our understanding of this complex disease has evolved considerably, progressing from a primarily genetic perspective to encompass various other facets, including the intricate alterations in cellular metabolism (2). Metabolic reprogramming in cancer cells, often termed as one of the ‘hallmarks of cancer’, has garnered significant attention due to its potential as a therapeutic tractable vulnerability (3). Tumors are far from being homogeneous entities, and the tumor microenvironment - an intricate network composed of cancer cells, immune cells, fibroblasts, blood vessels, and extracellular matrix - plays a vital role in tumor development, progression and metastasis (4). Crucially, the metabolic interactions within this complex network, often referred to as ‘metabolic crosstalk,’ significantly influence cancer progression and the therapeutic response (5). The exploration of these metabolic alterations not only improves our understanding of the disease but also holds the promise of identifying novel therapeutic vulnerabilities for cancer treatment.

In this special Research Topic, “*Cancer Metabolism: Molecular Insights, Metabolic Crosstalk in the Tumor Microenvironment, and Implications for Therapy*”, we have compiled a series of 23 articles that delve into the various aspects of cancer metabolism, highlighting both the potential therapeutic implications and the advancements in our understanding of metabolic alterations in cancer cells and in the tumor microenvironment. These articles explore the depth and breadth of cancer metabolism, examining metabolic pathways’ reprogramming in cancer cells, the interplay between tumor cells and the tumor microenvironment, and the implications for therapy (6). This editorial provides an overview of these insightful articles, showcasing the crucial contributions they make to this important area of research.

## Articles overview

The contributions to the Research Topic, “*Cancer Metabolism: Molecular Insights, Metabolic Crosstalk in the Tumor Microenvironment, and Implications for Therapy*,” cover various aspects of cancer metabolism. The fascinating realm of molecular biology has recently turned its lens to the mechanistic understanding of metabolic dysregulation displayed by cancer cells. As one of the most persistent and lethal diseases in human history, a thorough understanding of the cellular and molecular events that drive tumorigenesis, the disease aggressiveness and failure of therapy is critical. In this editorial, we bring together a series of recent studies that have each contributed unique insights into the metabolic dysfunctions that underlie cancer progression.

## Molecular mechanisms and metabolic dysregulation in cancer cells and stromal cells in tumor microenvironment

The research landscape on molecular mechanisms and metabolic dysregulation in cancer is brimming with new insights, painting a clearer picture of the intricate interplay among genes, proteins, and metabolic pathways. With every breakthrough, we grow closer to unlocking therapeutic strategies that can harness these discoveries. The contributions under this theme span a broad spectrum, from the enigmatic roles of mitochondrial uncouplers to the potentials of ferroptosis in glioma treatment. One seminal study unveils the paradoxical effects of the mitochondrial uncoupler 2,4-dinitrophenol (DNP) on human glioma cells. Contrary to conventional viewpoint, chronic exposure to DNP enhanced tumor growth and metastatic attributes, exhibiting elevated glycolysis and oxidative phosphorylation levels. These findings raise provocative questions, urging caution in the therapeutic use of mitochondrial uncouplers and advocating further probing into this conundrum (Rai et al.). Aboouf et al. offer a comprehensive analysis of erythropoietin receptor (EPOR) and its influence on cancer cell mitochondrial metabolism and tumor growth. By uncovering the mechanisms by which EPOR regulates mitochondrial biogenesis and metabolism, the study identifies novel paths for research into tumor growth modulation.

Like glucose, cancer cells are also addicted to enhanced glutamine uptake and metabolism. A study in this topic reevaluates role of enhanced glutamine metabolism and regulation of glutamine addiction in cancer. By unraveling the multifaceted relationship between glutamine and oncogenes, the review highlights the importance of polytherapeutic strategies targeting glutamine metabolism (Ni et al.). The study’s emphasis on the overlooked glutaminase II pathway brings a novel angle to glutamine metabolism’s targeting, casting a new perspective on therapeutic interventions

In a study by Wang et al., the role of inorganic pyrophosphatase (PPA1) in malignant tumors is meticulously evaluated. Recognized for its participation in energy metabolism, PPA1 emerges as a promising avenue for tumor diagnosis and therapy, with implications across various malignancies. Another key area of research in cancer and metabolism is the regulation of altered lipid metabolism, redox state and oxidative stress. Altered lipid metabolism can hamper the cellular redox state and can cause oxidative stress which can lead to cells death. Ferroptosis is a new form of regulated cell death characterized by iron-dependent lipid peroxidation. A review article published in this topic highlights the regulation of ferroptosis and its roles in glioma. The authors outline the key regulators, pathways, and crosstalk with other cell death forms, positioning ferroptosis as a potential target in glioma treatment. The insights into therapeutic resistance associated with ferroptosis pave the way for future treatment strategies in neuro-oncology (Shi et al.). Another review article under this topic provide an illuminating view of the Uridine diphosphate (UDP)-glycosyltransferases (UGTs) superfamily and its part in tumor cell metabolism. This article unravels the complex roles of UGTs in lipid, drug, and hormone metabolism, with far-reaching implications for cancer development and prognosis (Liu et al.).

A research article in this Research Topic investigated the role of chromatin regulators (CRs) on tumor microenvironment remodeling and hepatocellular carcinoma (HCC) prognosis. This study yields a groundbreaking CRsscore, serving as an independent prognostic index. The study sheds light on the intertwining of epigenetics, energy metabolism, and cuproptosis in HCC, opening new avenues for therapeutic targeting and personalized interventions (Dai et al.). Lastly, the work by Benito-Lopez et al. probes the relationship between metabolic reprogramming and immune checkpoints in the tumor microenvironment (TME). By uncovering how metabolic changes modulate anti-tumor immune functions, the review provides advancements in the understanding of the TME’s complexity. The insights into an immunosuppressive feedback loop steered by nutrient deprivation and by-product accumulation hold potential for synergistic cancer treatments with metabolic and immune checkpoint inhibitors.

Together, these contributions form a rich tapestry of cutting-edge research that elucidates the intricate molecular mechanisms at play in cancer metabolism. The pioneering studies featured here add invaluable dimensions to the growing knowledge base, strengthening the foundation for future therapeutic innovations and personalized patient care.

## Cancer metabolism as a therapeutic target: new insights and discoveries

Understanding the intricacies of cancer metabolism has become a crucial aspect of cancer research, heralding the discovery of innovative therapeutic targets and strategies. By harnessing the unique metabolic dependencies of cancer cells, researchers are opening doors to tailored treatments that promise more effective outcomes. This editorial delves into recent studies that have marked substantial progress in this field. Along with glucose metabolism, cancer cells also exhibit an altered lipid metabolism as it not only facilitates cancer cells to meet high energy demand but also help structural support such as biosynthesis of new plasma membrane (7). Taking this into consideration, targeting cholesterol metabolism could be an attractive therapeutic strategy for various cancer types. On these lines, a study spotlight the anti-tumor effects of stigmasterol, a plant-derived phytosterol, found in various plants such as herbs, soybeans, and tobacco. Not only is stigmasterol known for its anti-inflammatory and anti-diabetic properties, but it has also shown to have substantial anti-tumor activity in malignancies like breast, lung, liver, and ovarian cancers. Through mechanisms that include the regulation of the PI3K/Akt signaling pathway and effects on cyclins and cyclin-dependent kinases (CDKs), it demonstrates potential in promoting apoptosis, inhibiting proliferation, metastasis, and invasion, while inducing autophagy in tumor cells. Zhang et al.’s comprehensive review emphasizes stigmasterol’s promising role as a novel anti-tumor agent. Another study in this topic have taken an innovative prognostic approach by exploring fatty acid and lactate metabolism in osteosarcoma patients. Their research reveals that higher fatty acid and lactate risk scores may impair immune function and adversely affect patient prognosis. These findings could serve as a vital guide in shaping future osteosarcoma treatment strategies (Wu et al.).

The complex relationship between the tumor microenvironment (TME) and metabolic changes leading to drug resistance in cancer. An interesting study delve into studying the interaction between stromal and cancer cells in multiple myeloma (MM). By examining the interaction between MM cells and bone-marrow stromal cells (BMSCs), they uncover how the co-culture leads to a metabolic shift that favors drug resistance. Their work emphasizes the need for a more holistic approach to cancer biology, considering not only cancer cells but also their surroundings, which may reveal untapped therapeutic targets (Montoya et al.). Taking on the challenge of enhancing the effectiveness of STING agonists (STINGa) in cancer therapy, Moshnikova et al. present a pioneering method using pH Low Insertion Peptide (pHLIP). This approach extends the blood longevity of STINGa and targets them to acidic tumor components. The result is a remarkable potential to eradicate tumors and induce a sustained immune response against cancer recurrence. Moshnikova et al.’s study marks an essential development in cancer immunotherapy, shedding light on a strategy that not only targets tumors but also fortifies anti-cancer immunity by activating T-cells.

In conclusion, these recent studies collectively shed light on the dynamic landscape of cancer metabolism. From unveiling the potential of naturally occurring substances like stigmasterol to the innovative use of targeted peptides, the research pushes the boundaries of therapeutic intervention. By uncovering previously unexplored connections and emphasizing the complexity of metabolic network in cancer, they offer renewed hope for patients and practitioners alike. This fresh perspective on the metabolic aspects of cancer emphasizes the importance of continued research, collaboration, and innovative thinking in the relentless pursuit of better therapeutic outcomes.

## Exploring predictive models in cancer metabolism and their translational implications

The rising tide of computational biology and modeling is revolutionizing translational research, especially in the realm of cancer therapeutics. Through the prism of predictive models, we can decipher the intricate molecular facets of diseases and pave the way for innovative therapeutic interventions. In this editorial, we spotlight recent contributions under this research theme, highlighting the myriad ways predictive models and their translational applications in bolstering the fight against cancer. These diverse studies, though exploring multiple topics, converge on a shared mission: unraveling the multifaceted disease mechanisms to improve therapeutic outcomes in cancer patients.

In a notable investigation, Zhang et al. delved into metabolic shifts in clear cell renal cell carcinoma (ccRCC). Their goal was to construct a prognostic model to aid clinical endeavors. Through meticulous analysis, they pinpointed metabolic genes integral to ccRCC prognosis. This led to a prognostic risk score model with six pivotal genes showing a strong link with patient survival rates. The model’s efficacy was reaffirmed in multiple cohorts, emphasizing its potential in clinical prognosis and therapy for ccRCC. Li et al. presented a comprehensive meta-analysis on the proline metabolism enzyme Δ1-Pyrroline-5-carboxylate reductase (PYCR1) and its role in cancer prognosis. In a meta-analysis by collating data from diverse sources, they unveiled the correlation of high PYCR1 expression with increased tumor progression and metastasis in multiple cancer types. Cao et al.’s research casts light on fatty acid metabolism’s relationship with high-grade serous ovarian cancer (HGSOC) prognosis. Their study delineated a fatty acid signature correlating with prognosis, emphasizing the bridge between tumor microenvironment’s immune aspect and fatty acid metabolism. Liu et al.’s research emphasizes the potential of metabolites as pancreatic cancer biomarkers. They employed advanced techniques to discern metabolic changes in pancreatic cancer, revealing pathways crucial for tumor progression. Their findings advocate for the potential of these biomarkers in early diagnosis and prognosis prediction.

Jiang et al.’s comprehensive analysis offers fresh insights into the tumor microenvironment and metabolic attributes of colon adenocarcinoma. Their research underscores the value of immune- and metabolism-related genes in prognosis and therapeutic guidance. Tang et al.’s work delves into the role of lactate dehydrogenase A (LDHA) in T-cell responses against tumors. Their insights into LDHA’s functions and its modulation by epigenetic regulators illuminate the challenges and potential strategies for effective immunotherapy. Liu et al.’s study underscores the influence of lipid droplet metabolism and associated metabolic genes on gastric cancer. Their findings spotlight potential prognostic biomarkers and lay the groundwork for further investigative endeavors. Luo et al. evaluated the prowess of ^18^F-FDG PET/CT in diagnosing synchronous multiple primary malignant neoplasms. Their findings advocate for the diagnostic superiority of ^18^F-FDG PET/CT over conventional imaging for such malignancies.

Meng et al.’s exploration into mitochondrial metabolism-related genes offers a new perspective on high-grade serous ovarian cancer prognosis. Their findings emphasize the gene-tumor immune microenvironment interplay and its implications for future treatment strategies. Chang et al. introduces a groundbreaking prognostic gene signature linked to zinc metabolism in lung adenocarcinoma. Their work highlights the intricate relationship between zinc metabolism, tumor prognosis, and immunotherapeutic responses. Lastly, Du et al.’s article unveils the potential of metabolic long non-coding RNAs in predicting gastric cancer prognosis and immunotherapy efficacy. Their findings underscore the role of these lncRNAs in comprehending the disease’s immune landscape and in guiding therapeutic interventions.

These multifaceted studies illuminate the profound potential of predictive models and computational biology in enhancing our grasp of cancer metabolism. They offer exciting prospects for personalized treatment strategies, targeted therapeutics, and improved prognosis predictions. From biomarkers to enzyme studies and metabolic reprogramming, these articles collectively provide a comprehensive overview, setting the stage for future research and therapeutic innovation in the diverse and challenging landscape of cancer. The advancements herald a promising era where data-driven research insights translate into clinical realities, bringing us closer to the vision of precision medicine in oncology.

## Conclusions

The series of articles covered in this Research Topic underscore the incredible breadth and depth of ongoing investigations and discoveries in cancer metabolism. These studies and analyses collectively highlight how an improved understanding of metabolic pathways and their crosstalk within the tumor microenvironment can illuminate new therapeutic strategies. The intersection of metabolism with other key areas, such as immunology, epigenetics, and cell signaling, highlights the interconnectedness of various aspects of cancer biology. The incorporation of advanced technologies like machine learning models, artificial intelligence and NMR spectroscopy showcases the potential of integrating computational and experimental approaches in unraveling the complexities of cancer. Furthermore, the development of predictive models based on metabolic genes exemplifies the translational potential of these research findings. In conclusion, these studies signify a promising step forward in our quest to understand and to combat cancer. By harnessing the insights gained from these investigations, we move closer to develop effective therapeutic strategies that can exploit the metabolic vulnerabilities of cancer cells, thereby opening new avenues for precision oncology.

## Author contributions

BC: Conceptualization, Formal Analysis, Investigation, Resources, Supervision, Validation, Visualization, Writing – original draft, Writing – review & editing. PM: Conceptualization, Formal Analysis, Supervision, Validation, Visualization, Writing – review & editing.
